# A randomized controlled trial to test the efficacy of a brief Triple P discussion group to increase healthy feeding practices and reduce risk factors for infant obesity

**DOI:** 10.1093/jpepsy/jsae063

**Published:** 2024-09-09

**Authors:** Agnes Gelmini, Cassandra L Tellegen, Alina Morawska

**Affiliations:** Parenting and Family Support Centre, School of Psychology, The University of Queensland, St Lucia, Australia; Parenting and Family Support Centre, School of Psychology, The University of Queensland, St Lucia, Australia; Parenting and Family Support Centre, School of Psychology, The University of Queensland, St Lucia, Australia

**Keywords:** parenting, Triple P, infant obesity, randomized controlled trial

## Abstract

**Objective:**

Test the efficacy of a brief 2-hr parenting intervention in increasing protective factors against and reducing risk factors for infant obesity.

**Method:**

A 2 (Baby Healthy Living Triple P vs. care-as-usual) × 3 (baseline, postintervention, 6-month follow-up) design was used. Eighty-two parents of 4- to 18-month-old infants meeting at least two risk factors for early childhood obesity (e.g., parent/child overweight, low education level) were randomized to intervention (*n *=* *42) or control group (*n *=* *40). Parents questionnaires and child weight status was measured.

**Results:**

Results showed an intervention effect on a primary outcome, early feeding practices (restrictive: *d *=* *0.44, 95% CI [−0.01,0.88], pressuring: *d* = 0.11, 95% CI [−0.32,0.54], nonresponsive behaviors: (*d* = 0.32, 95% CI [−0.11,0.75]), and on a secondary outcome, feeding beliefs (*d* = 0.29, 95% CI [−0.14,0.73]). No beneficial impact was found on other primary outcomes (responsiveness in feeding: quantity *d *=* *0.50, 95% CI [−0.03,1.03]) and nutritive *d *=* *0.52, 95% CI [−0.03,1.07], mealtime environment: *d *=* *0.35, 95% CI [−0.78,0.08], self-efficacy in responsive feeding: *d *=* *0.21, 95% CI [−0.22,0.64]), or secondary outcomes (parental self-efficacy: *d *=* *0.08, 95% CI [−0.50,0.35]), parent emotional eating (*d *=* *0.01, 95% CI [−0.43,0.43]), food restraint (*d *=* *0.42, 95% CI [−0.85,0.02]), and body satisfaction (*d *=* *0.01, 95% CI [−0.43,0.43]) and child weight status (*d *=* *0.11, 95% CI [−0.54,0.32]).

**Conclusions:**

Promising though limited support was demonstrated for a brief, low-intensity program to help parents in the prevention of obesity for infants at risk.

The prevalence of overweight in childhood is a major public health issue (Lobstein et al., 2015). Recent estimates suggest that 9.2% of children under age 2 have weight-for-length (WFL) ratios ≥ 95^th^ percentile (Ogden et al., 2020). Infants with excess adiposity are at risk for short-term morbidities and in the long-term, poorer health trajectories and premature mortality (Caprio et al., 2020; Reilly & Kelly, 2011). To date, most preventative programs have targeted children between 2 and 11 years—an age at which obesity is likely to be already entrenched—with little success (Haynos & O’Donohue, 2012). Thus, infancy may be a critical period to intervene before children have well-established patterns of unhealthy eating and lifestyle habits (Pesch & Lumeng, 2021).

The etiology of obesity is multifactorial and complex, and several determinants of childhood obesity have been clearly identified, including modifiable risk factors such as diet, physical activity, or sleep duration (Aagaard et al., 2024). There is strong support for the notion that parental feeding practices are important in the development of infant overweight (DiSantis et al., 2011). For example, controlling feeding practices (e.g., pressuring or restrictive) have been associated with negative infant eating behaviors (e.g., tendency to overeat) and higher child weight status (Thompson, et al., 2013), while responsive feeding practices have been shown to positively impact child weight outcomes (Redsell et al., 2015). Furthermore, food preferences as well as problematic eating patterns acquired early in childhood are likely to track into adulthood (Dickens & Ogden, 2014). It has been suggested that diet and responsive feeding might be the most potent targets for parental behavioral change (Redsell et al., 2015), and addressing the barriers and enablers to assist responsive feeding is crucial (Redsell et al., 2021). Despite this evidence, few studies have specifically addressed parental feeding practices in preventative interventions during infancy, instead only targeting this later in development once feeding patterns are well established (Koplin et al., 2019).

Other factors have also been overlooked in previous research. Parental self-efficacy has been positively related to parental responsive feeding and child healthy eating yet has seldom been studied in infancy beyond the context of breastfeeding (Campbell et al., 2010; Koh et al., 2014). However, it has been recognized that strengthening parental behavioral self-efficacy around child feeding could increase the benefits of infant obesity preventative programs by fostering more durable changes in parental behaviors (Campbell et al., 2010; Redsell et al., 2015). Additionally, parent self-efficacy in general is a key intervention target for parenting programs and is often recognized to be a mechanism for change in parent and child outcomes (Sanders & Mazzucchelli, 2022).

The literature has also highlighted the critical role of the home and mealtime environments in the genesis of childhood obesity (Neumark-Sztainer et al., 2004; Smith et al., 2022). In the foundational years of infancy, children learn eating skills and other long-lasting lifestyle-related behaviors through mechanisms such as familiarization, observation and imitation of parents and significant others (Birch & Anzman, 2010). In that context, parental modelling has been noted as a key-factor (Birch & Doub, 2014; Dickens & Ogden, 2014) correlated with obesity-related outcomes such as children’s sedentary behaviors (Schrempft et al., 2015), television viewing (Campbell et al., 2013) and consumption of healthy or new foods (Draxten et al., 2014). In the family environment, parental behaviors and attitudes may impact through modelling on children’s health and the onset of obesity. For example, parents’ own nonresponsive eating behaviors (e.g., emotional or restrictive eating) have been shown to influence children’s eating patterns and have been associated with parental controlling feeding practices (Birch & Fisher, 1998). Similarly, parental body dissatisfaction has been correlated with early disordered eating in children (Fisher et al., 2009). However, although modelling seems to play a pivotal role, few studies (e.g., Walsh et al., 2014) have directly targeted parental modelling in infant obesity preventative programs and evidence of the influence of the mealtime environment in infancy is limited.

Collectively, these findings support the application of parenting interventions to address the prevention of infant obesity. However, despite the encouraging results of multi-component interventions (Daniels et al., 2012; Paul et al., 2016; Rossiter et al., 2021), feasibility in real-world settings in terms of costs, participant retention and delivery are questionable as programs are often long and intensive (e.g., six 2-hr sessions; Campbell et al., 2013) and reported as difficult to implement (Haynos & O’Donohue, 2012; Rossiter et al., 2021). This is particularly problematic for a population-wide issue such as obesity that requires cost-effective preventative programs for universal implementation (Laws et al., 2014). Given that previous research has demonstrated the efficacy of brief interventions in other domains (Sanders et al., 2014), the purpose of this study was to evaluate a low-intensity parenting intervention aimed at reducing risk factors associated with infant obesity.

A brief, multi-component, behavioral discussion group intervention, Baby Healthy Living Triple P (Morawska & Sanders, 2014), was developed for parents with babies at risk for overweight, which aimed to promote the formation of healthy habits and decrease risk factors associated with infant obesity. The Triple P-Positive Parenting Program is an evidence-based system of parenting interventions sitting based on a range of theories including social learning theory, social cognitive theory, self-regulation theory and developmental theory (Sanders et al., 2014). Triple P interventions aim to increase the skills, confidence and knowledge of parents in order to improve outcomes in families (e.g., increase positive parenting practices, decrease problems in children improve parent adjustment and relationships) (Sanders & Mazzucchelli, 2022). There are five levels of Triple P intervention including programs of differing lengths and intensity, delivery modality and target populations ranging from babies to children and adolescents and including special populations such as children with developmental disabilities. Baby Healthy Living Triple P is a low intensity (Level 3) intervention consisting of a 2-hr discussion group for a small group of parents. The specific objectives of this intervention are to increase the knowledge, skills and confidence of parents relating to infant feeding drawing on the literature around responsive feeding practices, mealtime environments and self-regulation of feeding. The content of this intervention is consistent with the commonly used behavior change techniques and theories for other existing interventions targeting obesity prevention in infancy (Matvienko-Sikar et al., 2019).

The aim of this study was to assess the efficacy of Baby Healthy Living Triple P in a randomized controlled trial. We expected that compared to the control group, parents in the intervention group would report greater improvements in the following parent-reported primary outcomes: an increase in responsive feeding practices, increase in positive mealtime environment, decrease in controlling feeding practices, and increase in self-efficacy in responsive feeding. We also predicted an intervention effect on the following parent-reported secondary outcomes: feeding beliefs, parental self-efficacy, parent emotional eating, food restraint and body satisfaction. Intervention effects were also predicted on the secondary outcome of child weight status based on measurements taken.

## Methods

### Participants

Participants were recruited through childcare services, playgroups, mothers’ groups, the University, health organizations and a baby sleep clinic in the Greater Brisbane region, including suburbs identified as most disadvantaged areas using the Index of Relative Socio-Economic Advantage and Disadvantage (Australian Bureau of Statistics, 2011). Advertising directed parents to the study website to complete online screening.

Parents eligible to participate in the study had a 4- to 18-month-old child, were interested in promoting their baby’s health and/or were concerned about feeding their child, and also met two or more risk factors associated in previous research studies with infant obesity: introduction of solid food before 4 months of age (Gibbs & Forste, 2014), parental overweight (Reilly et al., 2005), child overweight (Ong & Loos, 2006), sole parenthood (Morandi et al., 2012), low level of education (Gibbs & Forste, 2014), having the TV on during mealtimes (Schrempft et al., 2015). Parents were excluded if: currently receiving professional assistance for their child’s development or behavior, or psychological treatment for personal issues; their baby was born before 37 weeks gestation; their child had been diagnosed with a developmental delay or disorder; or parents were intellectually impaired.

Participants were 80 (97.6%) mothers and two (2.4%) fathers with a child aged between 4 and 18 months old. On average, parents were 32.4 years old (*SD *=* *4.15). Children were 44 girls (57%) and 38 boys (43%) and their mean age was 9.96 months (*SD *=* *4.38). Nine children (11%) were overweight, and one child was underweight. Nineteen babies (23.2%) were exclusively on a liquid diet (i.e., breastfed and/or formula-fed) and 63 (76.9%) had started on or were mainly fed solid food. Overall, 62.2% (*n *=* *50) of parents had overweight or obesity (35.4%, *n *=* *29 and 26.8%, *n *=* *21, respectively). Most parents (95.1%, *n *=* *78) were married or in a de facto relationship. Ethnic backgrounds were mainly Australian (86.6%, *n *=* *71) and European (6.1%, *n *=* *5). Most parents had a university degree (73.1%, *n *=* *60) and were working (84.1%, *n *=* *69) full time or part time. Nine parents (11%) reported not meeting their essential household expenses at a time over the last 12 months and 15 parents (18.3%) felt that they did not have enough money to buy much of anything they wanted.

### Measures

The *Family Background Questionnaire* (FBQ; Sanders & Morawska, 2010) gathered demographic information at preintervention. Height and weight were collected using professional Seca Aura 807 electronic scales and a Charder wall-mounted height stadiometer for adults, and Cupid 2 Baby electronic scales (to the nearest 0.01 kg) and a Seca 210 length mat (to the nearest 0.5 cm) for children up to 2 years old. All measurements were performed in duplicate to ensure accuracy and the mean of these measures was used. Parental body mass index (BMI; kg/m^2^) categories (World Health Organization, 2000) and infant WFL *z*-scores and percentiles (World Health Organization, 2006) were used.

#### Primary parent outcomes

**Early feeding practices.** The Infant Feeding Style Questionnaire (IFSQ; Thompson et al., 2009) is an 83-item measure of early maternal feeding practices validated among low-income African-American mothers of infants. It has multiple feeding style domains and subconstructs. We measured one or two subconstructs from each of the following styles: pressuring (Finish); restrictive: (Amount), and responsive (Satiety, Attention). Parents rated each question on a 5-point Likert-type scale ranging from 1 *(disagree)* to 5 (*agree*). In the present sample, these subconstructs showed acceptable internal consistency (.77, .72 and .71, respectively). In addition, as the IFSQ includes both beliefs and behaviors in each scale, we wanted to explore differences between these constructs. Therefore, feeding behavior items were averaged with reversed responsive items to provide a subscale of nonresponsive feeding behaviors (Cronbach’s *α* = .76). Similarly, and as part of our secondary outcomes, total mean scores for nonresponsive feeding beliefs items were calculated and used as a subscale (*α* = .61). Nonresponsive behaviors correlated strongly with nonresponsive beliefs, with a Pearson’s *r* = 0.61, *p* < .001.

**Responsiveness in feeding.** The *Mealtime Scenarios* (MealS; Gelmini & Morawska, 2014b) parental responsiveness scale was developed to complement the IFSQ. Each question consists of a brief vignette followed by two questions: (1) *what do you do in this situation?* And (2) *why do you react that way?* Assessors blind to condition and time used a protocol to code responses. Both responses were used to evaluate the degree of responsiveness in parents’ answers, which was rated on a 9-point Likert-type scale (1 = *very responsive*, 9 = *very controlling*). Responsive feeding behaviors are based on a belief that the child can self-regulate food intake and having an intention to let them regulate themselves. Examples of responsive feeding behaviors are the parent feeding the child according to appetite cues because they believe their child knows when they are hungry or full, not offering other food instead if the child refuses to eat or eats very little or letting the child decide what to eat. Controlling feeding behaviors are based on not believing that the child can self-regulate food intake and having no intention to let them regulate themselves. Examples include insisting and pressuring the child to eat even if they show signs of being full, offering several different foods even when the meal is over if the child refuses to eat, and using the TV or other distractions so the child eats enough. Scores were then reversed so that a high score represents more responsive feeding. In scenario 1, a child refuses to eat more than a few mouthfuls at the dinner table. In scenario 2, a child eats a lot of one food (i.e., meat) and leaves out the other foods (i.e., vegetables). Scenario 1 captured parental responsiveness related to the quantity of food eaten by the child (Quantity), while scenario 2 captured parental responsiveness related to the nutritive quality of food eaten by the child (Nutritive). If parents considered that the scenarios did not apply to them, they answered *not applicable* (NA). To assess interrater reliability, 25% of the overall MealS data was coded by two assessors; the intra-class correlation coefficient was *α* = .78 for Quantity, and *α* = .95 for Nutritive, showing good interrater reliability.

**Mealtime environment.** The *Family Lifestyle Scale* (FLS; Gelmini & Morawska, 2014a) is a 19-item scale with a 5-point rating from *never* (1) to *most of the time* (5) developed for the present study to measure family lifestyle behaviors, with a main focus on the mealtime environment (13 items). Mealtime environment questions captured three areas of functioning: (i) Atmosphere, based on the Positive Mealtime Environment factor from the About Your Child’s Eating questionnaire (Davies et al., 2007); (ii) Structure, adapted from a study on family mealtime behaviors (Wansink & Kleef, 2014) and; (iii) Modelling, adapted from research by Neumark-Sztainer et al. (2004). Example items for the three subscales are as follows: *we have nice conversations during meals, the TV is switched on during mealtimes*, and *I try to set a good example at the table so that my child learns how to behave as they grow up.* These three areas were collapsed into a Mealtime Environment (ME) factor for analysis, which had good internal consistency (*α* = .83).

**Self-efficacy in responsive feeding.** The *Anxiety and Confidence Scale* (ACS; Gelmini & Morawska, 2013) was developed to capture parental anxiety and self-efficacy related to letting their baby self-regulate their feeding. In 6 items, parents were asked to rate how confident and anxious they felt in three mealtime situations, from *extremely* (1) to *not at all* (5). An item example was *how confident/anxious do you feel about letting your baby decide how much they want to eat at meal/feeding time?* Higher total scores indicated greater self-efficacy (anxiety items reversed) and good internal consistency was found (*α* = .79).

#### Secondary parent outcomes

**Feeding beliefs.** Parental beliefs about feeding were measured using the IFSQ.

**Parental self-efficacy.** The *Maternal Self-Efficacy Scale* (MSES; Teti & Gelfand, 1991) is a 10-item scale assessing general maternal self-efficacy and behavioral competence among mothers of 3- to 13-month-old infants on a 4-point scale rating from 1 (*not good at all*) to 4 (*very good*). Items assessed self-efficacy for a range of tasks including soothing, understanding baby, and having fun with baby. The MSES showed good internal consistency (*α* = .81). This measure of general self-efficacy was included to assess the extent to which any improvements in self-efficacy would generalize beyond the feeding domain.

**Parent emotional eating, food restraint, and body satisfaction.** These three constructs were measured using three items from the Revised Three-Factor Eating Questionnaire (Karlsson et al., 2000). One item evaluated emotional eating on a 4-point scale (1 being *definitely false* to 4 being *definitely true*) the following statement: *when I feel anxious and blue, I find myself eating.* The second item assessed Cognitive Restraint on food and was rated on an 8-point scale from *eat whatever/whenever I want* (1) to *constantly limiting food intake* (8). The third item (*I am satisfied with the appearance, size and shape of my body*) with an 8-point rating from 1 (*I strongly agree*) to 8 (*I strongly disagree*), addressed parental body satisfaction.

#### Secondary child outcome

**Children’s weight status.** This was measured as described above.

#### Parent satisfaction

The *Client Satisfaction Questionnaire* (CSQ; Sanders et al., 2001) is a 6-item instrument completed at postintervention that evaluates parental satisfaction of the intervention on a 7-point rating scale from 1 (*poor*) to 7 (*excellent*). The internal consistency for the CSQ was excellent (*α* = .92).

### Design

The study was a randomized controlled trial with two parallel groups (intervention versus control) using a repeated measures design (three time points: pre, post and 6-month follow-up). The allocation ratio was 1:1. All measures were completed online at three time points, except for FBQ only at preintervention, and the parent satisfaction measure only completed at postintervention. The control group was care-as-usual and parents were not contacted between pre- and postassessment but were free to access other supports.

### Procedures

The Consolidated Standards of Reporting Trial (CONSORT) were followed, and the final checklist is available as Supplemental File 1. Ethical clearance was granted from the Behavioural and Social Sciences Ethical Review Committee of The University of Queensland. The trial was registered with the Australian New Zealand Clinical Trials Registry (ACTRN12614001010684). All participants provided online informed consent. Data are available on request.

#### Randomization and process pathway

Parents who met eligibility criteria consented on the study website and completed the online preintervention assessment, then randomization occurred during the first home visit. The first author did home visits to take anthropometric measures after completion of the questionnaires at each time point. A person independent of the project used a computer-generated list of random numbers to prepare group allocation letters which were concealed from both researchers and participants in sealed envelopes, labeled with sequential numbers. At the initial home visit, the first author implemented the allocation to group by sequentially giving an envelope to the parents assigning them to group. Parents in the intervention group attended the program as soon as a minimum number of parents could attend a session (on average 31 days) and completed the second questionnaire 4 weeks after intervention (on average 36 days). Parents in the control group waited 6 weeks (47 days on average) after randomization before completing time 2 assessment. On average, the time delay between pre- and postintervention assessment for both groups was 55 days (*M *=* *55.17, *SD *=* *20.88). Both groups completed time 3 assessment 6 months postrandomization (186 days on average) (*M *=* *186.26, *SD *=* *27.46). After follow-up, parents in the control condition were offered to attend the program or a similar program more suitable for toddlers or view the recording of a group.

#### Intervention

Baby Healthy Living Triple P (Morawska & Sanders, 2014) is a brief parenting discussion group underpinned by a behavioral and social learning approach to family intervention, aimed primarily to empower parents in using responsive feeding practices to foster their child’s self-regulation of eating and to model healthy habits from the start. The proposed mechanisms of change of the intervention are to increase the knowledge, skills and confidence of parents relating to infant feeding to impact on responsive feeding practices (e.g., following children’s hunger cues), setting up the mealtime environment and feeding self-efficacy, as well as feeding beliefs, parent eating behaviors and general self-efficacy. Additionally, a secondary aim of the intervention would be for changes in parent outcomes to lead to improvements in child weight status over time. The program consisted of one 2-hr discussion group with the following sections: the challenges of raising a healthy baby, including the rise of infant obesity; infant self-regulation of energy intake and child-led feeding; healthy physical activity habits; infant sleep; role modelling as an early teaching strategy; positive parenting strategies (e.g., praise, backing up instructions) and coping skills to deal with emotions (e.g., anxiety). Group sessions were designed to be interactive, whereby each section included information giving and questions/points to generate discussion and input from parents on the topic. The facilitator worked through the sections in order using presentation slides and parents received a workbook summarizing the content delivered and with room to make notes based on the discussion points. After the intervention, participants received two generic follow-up emails encouraging them to implement strategies. Intervention sessions were held between September 2014 and June 2015 at the University of Queensland in Brisbane, Australia and facilitated by the first author (accredited Triple P practitioner). Both parents from two-parent families were invited to attend and an average of six parents (*M *=* *6.00, *SD* =1.58) from three families (*M *=* *3.23, *SD* =1.01) attended each group with 13 sessions conducted in total.

#### Protocol adherence

All 13 intervention sessions were videotaped. Structured checklists detailing the content to be covered in each section in order (e.g., child led feeding, role modelling, praise), were completed for each session by the practitioner and coded for protocol adherence. An independent coder (research assistant familiarized with the intervention) rated 25% of the recorded sessions (4 sessions). Agreement rate between the practitioner and the coder was 100% and 100% of the session content was covered in all 13 sessions.

### Statistical analyses

A priori power analyses (using G Power software) suggested a sample size for the present study of 111, assuming 80% power and a small effect size (*d *=* *0.30) based on estimates of a previous brief Triple P parenting intervention (Morawska & Sanders, 2011). Preliminary analyses compared intervention versus control groups at preintervention. A multivariate repeated measures analysis of variance (MANOVA) tested the effect of the intervention on conceptually related variables for feeding practices. Univariate ANOVAs were used to assess other primary outcomes. A series of ANOVAs and Chi-square tests (using Fisher’s exact test to calculate exact *p* values when Chi-square assumption of a minimum of five frequencies per cell was violated) assess secondary outcomes.

An intent-to-treat approach was used with Expectation Maximisation used to impute missing values (both from missing assessment items and attrition of participants) (Dempster et al., 1977). To increase the statistical power of the scales, imputation was performed at item-level rather than scale-level (Gottschall et al., 2012). All missing values were imputed except for the qualitative data of the MealS. Effect size estimates were calculated with Cohen’s *d* based on the mean pre-post or pre- follow-up change in the intervention group minus the mean pre-post or pre- follow-up change in the control group, divided by the pooled preintervention standard deviation, which is recommended for controlled group designs with repeated measures (Morris, 2007).

## Results

### Preliminary analyses

There were 6.3% and 0.03% missing values in the datasets for the intervention group and the control group, respectively. Little’s Missing Completely at Random (MCAR) test revealed that the data were missing completely at random for the intervention group, *χ*^2^(620) = 141.35, *p* > .999, and for the control group, *χ*^2^(737) = 0.000, *p* > .999. Because of the qualitative nature of the MealS data, missing values from that instrument were not included in the analyses of missing data reported above but were examined separately. Analyses showed that there was 4% and 0% of missing data in the intervention and control groups, respectively, and 11.4% of uncodeable data (i.e., *not applicable* answers), of which 7.5% occurred at time 1. Little’s MCAR indicated that the MealS data were missing at random, *χ*^2^(46) = 39.49, *p* = .740. In the overall dataset of this study, less than 0.05% of missing data was due to reasons other than attrition.

There was only one significant difference between groups at baseline. Infants in the intervention group had significantly higher WFL percentiles than infants in the control group at baseline and there was a significant difference between groups in the proportions of children in the weight status categories (see Table 1).

**Table 1.** Demographic characteristics of the intervention and control groups.

Variable	Intervention (*n *=* *42)		Control (*n *=* *40)			
Continuous	*M*	*SD*	*M*	*SD*	*t*	*p*
Child age (months)	10.17	4.70	9.90	4.08	0.38	.706
Parent age (years)	32.48	4.03	32.33	4.31	0.16	.870
Child’s WFL percentiles	65.35	27.27	52.42	28.44	2.10	.039*

**Categorical**	***n***	**%**	***n***	**%**	***χ²***	***p***

**Child sex**					0.42	.517
Male	18	42.9	20	50		
Female	24	57.1	20	50		
**Parent sex**					1.95	.162
Male	2	4.8	0	0.0		
Female	40	95.2	40	100		
**Child WFL percentile category**					6.47	.039*
Underweight (<5)	0	0.0	1	2.5		
Normal weight (5–94.9)	29	69	32	80		
Overweight (>95)	8	19	1	2.5		
**Parent weight category^a^**					0.18	.980
Underweight (BMI <18.5)	2	4.9	2	5.1		
Healthy weight (BMI 18.5–24.9)	14	34.1	12	30.8		
Overweight (BMI 25–29.9)	14	34.1	15	38.5		
Obese (BMI >30)	11	26.8	10	25.6		
**Martial status**					0.01	.960
Married/de facto	40	95.2	38	95		
Single/Divorced/Separated	2	4.8	2	5		
**Child ethnicity**					1.50	.827
Caucasian Australian	35	83.3	36	90		
European	3	7.14	2	5		
Sub-Saharan Africa	1	2.38	0	0		
North-East Asian	1	2.38	1	2.5		
South-East Asian	2	16.7	1	10		
**Parent education**					2.05	.358
High school	4	9.5	5	12.5		
Diploma	9	21.4	4	10		
University degree	29	69.1	31	77.5		
**Parent employment**					1.85	.397
Full-time	20	47.6	16	40		
Part-time	14	33.3	19	47.5		
Unemployed	8	19	5	12.5		
**Meeting household expenses**					0.07	.783
Yes	37	88.1	36	90		
No	5	11.9	4	10		
**After expenses can afford**					1.54	.464
Most things	18	42.9	21	52.5		
Some things	17	40.5	11	27.5		
Not much	7	16.7	8	20		
**Family structure**					0.27	.604
Original family	40	95.2	37	92.5		
Other (e.g., sole parent, extended)	2	4.8	3	7.5		

*Note*. BMI = body mass index; WFL = weight for length.

a *n *=* *80. Two mothers excluded from BMI calculation due to pregnancy.

* *p* < .05.

#### Attrition

**Figure 1.**
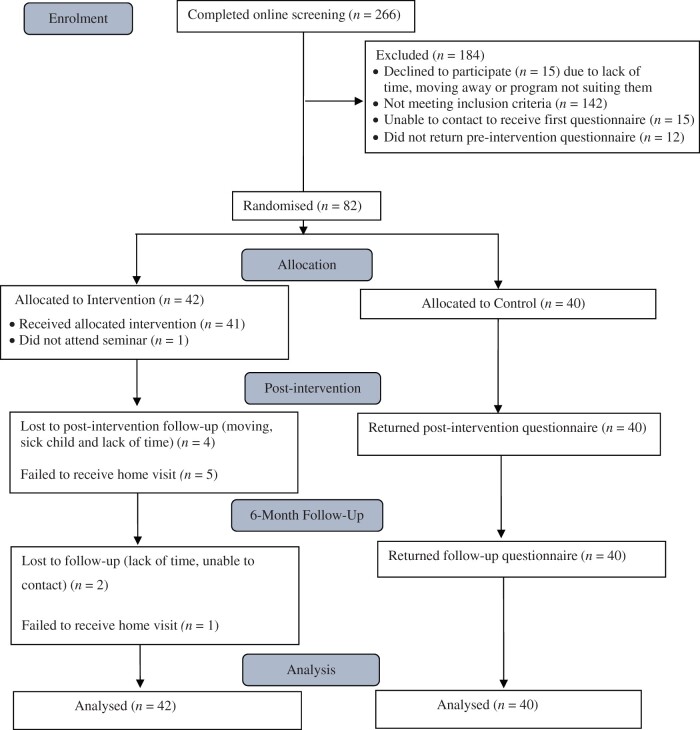
CONSORT diagram of flow of participants.

Figure 1 shows the flow diagram for this study according to the CONSORT. Of 266 people who completed online screening, 142 (54.4%) did not meet eligibility criteria and a further 15 could not be contacted with another 15 declining participation. Ninety-four parents consented to participate however 12 withdrew before randomization, leaving 82 parents randomized.

Retention rates were 95% at time 2 and 93% at time 3 with all dropouts from the intervention group. Chi-square tests indicated that the difference in attrition rates was not significant at postintervention, *χ*^2^(1, 82) = 2.2, *p* = .116, but was significant at follow-up, *χ*^2^(1, 82) = 4.24, *p* = .026. There were no significant differences between completers and noncompleters on demographic or outcome variables.

### Intervention effects

#### Primary outcomes

The descriptive statistics and time by condition interaction effects are reported in Table 2.

**Table 2.** Time by condition interaction effects.

Intervention (*n *=* *42)^a^			Control (*n *=* *40)^b^								
Pre	Post	FU	Pre	Post	FU			T1–T2		T1–T3	
*M* (*SD*)	*M* (*SD*)	*M* (*SD*)	*M* (*SD*)	*M* (*SD*)	*M* (*SD*)	*F* (*df*)	*p*	*d*	95% CI	*d*	95% CI
											
2.01 (0.67)	1.90 (0.58)	1.68 (0.47)	2.10 (0.63)	2.19 (0.76)	2.18 (0.73)	9.02 (2, 160)	<.001^**^	0.44	−0.01,0.88	0.76	0.32,1.21
2.30 (1.13)	2.05 (0.98)	1.73 (0.68)	2.33 (0.80)	2.19 (0.94)	2.21 (0.92)	3.31 (2, 160)	.039^*^	0.11	−0.32,0.54	0.45	0.02,0.89
4.46 (0.46)	4.63 (0.36)	4.70 (0.30)	4.34 (0.50)	4.42 (0.41)	4.44 (0.40)	1.09 (2, 160)	.338	0.19	−0.24,0.62	0.29	−0.14,0.72
2.01 (0.57)	1.81 (0.47)	1.63 (0.43)	2.05 (0.48)	2.02 (0.48)	2.04 (0.50)	8.62 (2, 79)	<.001^**^	0.32	−0.11,0.75	0.69	0.25,1.14
3.87 (2.77)	5.29 (3.24)	4.83 (2.60)	3.22 (3.14)	3.12 (2.74)	4.66 (2.87)	1.85 (2, 53)	.167	0.50	−0.03,1.03	0.16	−0.68,0.36
4.73 (2.80)	6.32 (2.77)	7.45 (2.22)	3.83 (3.33)	3.77 (2.75)	4.83 (3.23)	2.78 (2, 49)	.072	0.52	−0.03,1.07	0.54	−0.01,1.09
3.22 (0.79)	3.81 (0.68)	3.83 (0.57)	3.17 (0.79)	3.48 (0.84)	3.55 (0.70)	2.08 (2, 79)	.132	0.35	−0.78,0.08	0.29	−0.72,0.14
2.29 (0.75)	1.93 (0.63)	1.71 (0.51)	2.50 (0.77)	2.30 (0.78)	2.22 (0.78)	2.59 (2, 79)	.082	0.21	−0.22,0.64	0.39	−0.04,0.82
											
1.76 (0.61)	1.58 (0.57)	1.39 (0.37)	1.86 (0.53)	1.85 (0.68)	1.80 (0.68)	3.32 (2, 79)	.041^*^	0.29	−0.14,0.73	0.54	0.10,0.97
3.24 (0.42)	3.30 (0.38)	3.38 (0.35)	3.30 (0.37)	3.33 (0.36)	3.34 (0.34)	1.12 (2, 79)	.333	0.08	−0.50,0.35	0.25	−0.68,0.18
2.60 (0.86)	2.55 (0.85)	2.44 (0.75)	2.60 (0.78)	2.55 (0.93)	2.55 (0.96)	0.34 (2, 79)	.715	0.01	−0.43,0.43	0.13	−0.30,0.56
3.52 (1.29)	3.68 (1.67)	3.44 (1.44)	3.88 (1.27)	3.50 (1.40)	3.35 (1.27)	1.30 (2, 79)	.280	0.42	−0.85,0.02	0.35	−0.78,0.08
3.74 (2.05)	3.81 (2.31)	4.10 (2.22)	4.38 (1.79)	4.45 (1.91)	4.45 (1.85)	0.51 (2, 79)	.600	0.01	−0.43,0.43	0.15	−0.58,0.28
											
0.59 (1.03)	0.81 (0.96)	0.94 (0.90)	0.07 (0.92)	0.18 (0.89)	0.46 (0.75)	1.17 (2, 79)	.317	0.11	−0.54,0.32	0.04	−0.39,0.50

*Note*. ACS = Anxiety and Confidence Scale; FLS = Family Lifestyle Scale; IFSQ = Infant Feeding Style Questionnaire; MealS = Mealtime Scenarios; MSES = Maternal Self-Efficacy Scale; WFL = weight-for-length.

a *n *=* *24 for responsive quantity and *n *=* *22 for responsiveness nutritive (pairwise exclusions due to uncodeable data).

b *n *=* *32 for responsive quantity and *n *=* *30 for responsiveness nutritive (pairwise exclusions due to uncodeable data).

* *p* <.05.

** *p* <.001.

**Early feeding practices.** The repeated-measures MANOVA showed a significant time by condition interaction on parental feeding practices (pressuring Finish, restrictive Amount, responsive Satiety), using Wilks’ criterion, *F*(6, 75) = 3.07, *p* = .010. Univariate analyses revealed that parents in the intervention group reported significantly less pressuring and restrictive feeding than parents in the control condition, with a medium effect size at time 2 (*d *=* *0.44, 95% CI [−0.01,0.88]) and a large effect size at time 3 (*d* = 0.76, 95% CI [0.32,1.21]) for the pressuring practices and a small effect size at time 2 (*d* = 0.11, 95% CI [−0.32,0.54]) and a medium effect size at time 3 (*d* = 0.45, 95% CI [0.02,0.89]) for restrictive feeding practices. Parents in the intervention group reported significantly less nonresponsive behaviors than the controls, at postintervention with a medium effect size (*d* = 0.32, 95% CI [−0.11,0.75]) and at follow-up with a medium to large effect size (*d* = 0.69, 95% CI [−0.25,1.14]). There was no significant intervention effect for responsive satiety, (*d* = 0.19, 95% CI [−0.24,0.62]).

**Responsiveness in feeding.** There was no significant intervention effect for responsiveness (mealtime scenarios) Quantity (*d *=* *0.50, 95% CI [−0.03,1.03]) or Nutritive (*d *=* *0.52, 95% CI [−0.03,1.07]).

**Mealtime environment.** There was no significant intervention effect on the mealtime environment variable (*d *=* *0.35, 95% CI [−0.78,0.08]).

**Self-efficacy in responsive feeding.** There was no significant intervention effects on self-efficacy in responsive feeding (*d *=* *0.21, 95% CI [−0.22,0.64]).

#### Secondary outcomes

**Feeding beliefs.** There was a significant intervention effect on nonresponsive feeding beliefs (Finish, Amount and reversed Satiety beliefs items). Parents in the intervention group reported significantly less nonresponsive feeding beliefs than the controls, with a small effect size at postintervention (*d* = 0.29, 95% CI [−0.14,0.73]) and a medium effect size at follow-up (*d* = 0.54, 95% CI [0.10,0.97]).

**Parental self-efficacy.** There was no significant intervention effect for parental self-efficacy (*d *=* *0.08, 95% CI [−0.50,0.35]).

**Parent emotional eating, food restraint, and body satisfaction.** There were no significant intervention effects for emotional eating (*d *=* *0.01, 95% CI [−0.43,0.43]), cognitive restraint of food (*d *=* *0.42, 95% CI [−0.85,0.02]), and body satisfaction (*d *=* *0.01, 95% CI [−0.43,0.43]).

**Children’s weight status.** There was no significant difference in the proportion of underweight, healthy weight and overweight infants between groups at postintervention, *χ^2^*(2, 82) = 2.59, *p* = .279, nor at follow-up, *χ*^2^(2, 82) = 2.88, *p* = .266. There was also no significant time by condition interaction in WFL z-scores (*d *=* *0.11, 95% CI [−0.54,0.32]).

#### Parent satisfaction

Parents in the intervention group were overall satisfied with the program (*M *=* *5.66; *SD *=* *0.97) and highly satisfied with the quality of the delivery (*M *=* *6.16; *SD *=* *0.79). Parents reported that they generally received the type (*M *=* *5.21; *SD *=* *1.21) and amount (*M *=* *5.45; *SD *=* *1.06) of information they expected and that most of their needs were met (*M *=* *5.13; *SD *=* *1.32). They also indicated that they would seek help from Triple P again if needed (*M *=* *5.61; *SD *=* *1.10).

## Discussion

This randomized controlled trial examined whether a brief behavioral preventative intervention for parents of infants at risk for obesity increased effective parenting practices and reduced risk factors for early childhood obesity. Results showed that the intervention had a positive influence on the primary target, parental feeding practices: parents reported being less likely to exert pressure on or to restrict their child’s eating and to display controlling feeding behaviors; and on a secondary outcome, parents were less likely to report nonresponsive feeding beliefs. No significant intervention effects were found on parent-reported responsive feeding practices, mealtime environment or parent self-efficacy in responsive feeding, nor on the secondary outcomes of parental self-efficacy, parent-reported emotional eating, food restraint and body satisfaction, and children’s weight status.

Regarding parents’ feeding practices, our findings are partially congruent with results from one of the rare interventions (i.e., NOURISH; Daniels, et al., 2012) that have explicitly reported effects on parental responsive and controlling feeding practices. Similar to Daniels et al. (2012), we demonstrated a decrease in parental controlling feeding due to the intervention. However, while they found positive behavior changes in responsive feeding, we did not. This lack of interaction effect may be due to a lack of power, since pairwise exclusions used in the MealS analyses reduced the power considerably. An intervention effect was found on parental cognitions (i.e., decrease in nonresponsive feeding beliefs). Overall, a diminution of nonresponsive beliefs around feeding seems to align with the significant and enduring reduction in controlling feeding practices observed in the parents who attended the intervention.

There was no significant intervention effect on parental behaviors around mealtime, which may be related to the age of the children. While impacts of the mealtime environment have been studied in older child populations (e.g., Neumark-Sztainer et al., 2004), it has not been previously studied with infants. Parents with infants may find these changes more difficult to implement or may not perceive these changes to be as relevant while the children are young. However, even at an early age, family meal patterns and broader eating context play a critical role for the development of children’s eating habits and obesity (Pesch & Lumeng, 2021; Smith et al., 2022). Further investigation of the different components of the mealtime environment is warranted to tailor strategies to parents with infants.

In the broader eating context of the home environment, we also examined parents’ body satisfaction and eating behaviors (i.e., emotional and restrictive eating) because of their potential relationship with risk factors for infant obesity such as parental nonresponsive feeding practices (Fisher et al., 2009) and the role they may play in the emergence of childhood overweight (Aagaard et al., 2024). Even though those parent-related variables were not directly targeted in our intervention, we nevertheless expected that they might shift positively as an indirect consequence of parental empowerment. Results, however, did not reveal any change on parental eating behaviors and body satisfaction, which suggests that a specific approach may be required to address those factors. Future research should assess the potential benefit of incorporating such parent-focused components in preventative interventions.

There was no significant effect of the intervention on self-efficacy in feeding or general self-efficacy, which was surprising given one goal of the intervention was to empower parents. The feeding self-efficacy measure was new but did mix items with feelings of anxiety or worry in feeding which may have been measuring a different construct which may not have been addressed in the intervention. The MSES is a well-used self-efficacy measure which had good internal consistency in this sample (consistent with prior research; Teti & Gelfand, 1991). However, the average scores for general self-efficacy were quite high, meaning ceiling effects could have impacted on the results. There was no significant intervention effect on children’s weight status. Although this result contradicts our hypothesis, it appears congruent with systematic review evidence (Redsell et al., 2015) showing that few infant obesity prevention programs have an impact on child growth and even less on infant overweight prevalence, with the main effects being found on parental behaviors. Importantly, this intervention was only one brief 2-hr discussion group. It is likely that more intensive interventions over longer periods of time are needed in order to see change in a number of the outcomes measured in this study, in particular, weight status.

This study found a ratio of two times more responsive than nonresponsive feeding practices found in the sample, which is contradictory to several studies that have showed high levels of feeding control in parents of infants (Thompson et al., 2009). It is possible that parents underestimated their controlling feeding practices because coaxing young children to eat more is widely normalized (Birch & Doub, 2014). Parents may poorly recognize pressure or restriction when they exert it on their child’s eating, which could explain the low rates of controlling feeding reported overall. This sample may have had higher levels of responsive feeding, as they are likely to have also had access to information and education on feeding, as well as social opportunity, advice and support which are all identified enablers to responsive feeding (Redsell et al., 2021).

Study strengths included a randomized controlled trial with a repeated measures design, high retention and response rates. We also addressed a variety of constructs rarely investigated in previous studies (e.g., self-efficacy around feeding, mealtime environment, parental self-efficacy). However, it should be noted that all but the measure of child weight were parent-report questionnaires. Future research would benefit from the inclusion of more objective measures, less subject to social desirability bias, such as observational approaches. A lack of reliable measures was one of the most significant limitations of our study. Previous intervention trials that were successful in improving child weight outcomes and/or parental feeding practices (Redsell et al., 2015), assessed feeding practices using measures that differed from one trial to another (e.g., Campbell et al., 2013). Selecting reliable measures that would be appropriate for an infant population was challenging. Because our primary measure (i.e., IFSQ) had been validated for specific subgroups of the population and had some important conceptual (e.g., including both belief and behavior items in the same scales) and methodological issues (Wood et al., 2016), we developed a complementary measure for parental feeding practices, the MealS. However, significant change was not found on the MealS (likely influenced by lack of power) and the MealS also requires improvement of the wording, further validation and exploration of the other constructs assessed by the measure, as well as consideration of the developmental appropriateness to use this measure across a wide infant age range. One of the methodological limitations with the IFSQ was the absence of clinical cutoffs and published test–retest reliability that rendered the calculation of reliable change indices impossible, hence results must be interpreted with caution. Additionally, the nonresponsive feeding beliefs subscale was calculated for the purpose of this research and despite showing significant change, had a low alpha value, only just considered acceptable. Overall, further validation work is needed on a number of the measures used so that these important constructs can be accurately measured in early childhood obesity prevention research. Some recent work trying to establish what core outcomes to measure in infant obesity prevention work has laid a good foundation for further work in this area (Brown et al., 2022).

The study sample included a large age range of infants from 4 to 18 months. Having interventions that can be used across large age ranges is beneficial for research recruitment and dissemination purposes, however, the developmental changes which occur during these ages is substantial. This age group may be too heterogenous for inclusion in the same intervention with specific strategies being difficult to apply across infants, infants, although we did not experience difficulties with this during the intervention delivery. Another limitation of the sample was the overall high educational level and financial comfort of our sample that limits the generalizability of the results to higher risk communities in particular (e.g., low socioeconomic backgrounds, Indigenous families). However, it is worth noting that despite our sample being demographically at low risk for obesity, recruitment was limited to parents who were concerned about feeding or wanted information about promoting infant health and met at least two risk factors (however risk factors were not specifically tracked or analyzed). Parental overweight rates were still consistent with those in the Australian population, suggesting the screening method was successful in recruiting our target population of infants at risk for obesity. The fact that the intervention showed some positive effects on infant obesity-related determinants in a fairly homogeneous high SES sample is encouraging, especially given that the study was also underpowered. Future research should be conducted on populations at higher risk for early childhood overweight and with a focus on tracking risk factors and investigating impacts of interventions on specific risk factors.

## Supplementary Material

jsae063_Supplementary_Data

## Data Availability

Data is available on request.

## References

[jsae063-B1] Aagaard K. M. , Barkin S. L., Burant C. F., Carnell S., Demerath E., Donovan S. M., Eneli I., Francis L. A., Gilbert-Diamond D., Hivert M.-F., LeBourgeois M. K., Loos R. J. F., Lumeng J. C., Miller A. L., Okely A. D., Osganian S. K., Ramirez A. G., Trasande L., Van Horn L. V., Yanovski S. Z. (2024). Understanding risk and causal mechanisms for developing obesity in infants and young children: A National Institutes of Health workshop. Obesity Reviews: An Official Journal of the International Association for the Study of Obesity, 25(4), e13690. https://doi.org/ 10.1111/obr.13690 38204366 10.1111/obr.13690

[jsae063-B2] Australian Bureau of Statistics. (2011). Census of Population and Housing: Socio-Economic Indexes for Areas (SEIFA) (cat. no. 2033.0.55.001). Australia.

[jsae063-B3] Birch L. L. , Anzman S. L. (2010). Learning to eat in an obesogenic environment: A developmental systems perspective on childhood obesity. Child Development Perspectives, 4(2), 138–143. 10.1111/j.1750-8606.2010.00132.x

[jsae063-B4] Birch L. L. , Doub A. E. (2014). Learning to eat: Birth to age 2 y. The American Journal of Clinical Nutrition, 99(3), 723S–728S. 24452235 10.3945/ajcn.113.069047

[jsae063-B5] Birch L. L. , Fisher J. (1998). Development of eating behaviors among children and adolescents. Pediatrics, 101(3 Pt 2), 539–549. 12224660

[jsae063-B6] Brown V. , Moodie M., Sultana M., Hunter K. E., Byrne R., Seidler A. L., Golley R., Taylor R. W., Hesketh K. D., Matvienko-Sikar K. (2022). Core outcome set for early intervention trials to prevent obesity in childhood (COS-EPOCH): Agreement on “what” to measure. International Journal of Obesity (2005), 46(10), 1867–1874. 10.1038/s41366-022-01198-w 35927469 PMC9492532

[jsae063-B29265765] Campbell K., , Hesketh K., , Silverii A., & , Abbott G. (2010). Maternal self-efficacy regarding children’s eating and sedentary behaviours in the early years: associations with children’s food intake and sedentary behaviours. International Journal of Pediatric Obesity: IJPO: An Official Journal of the International Association for the Study of Obesity, 5(6), 501–508. 10.3109/17477161003777425 20429735

[jsae063-B7] Campbell K. J. , Lioret S., McNaughton S. A., Crawford D. A., Salmon J., Ball K., McCallum Z., Gerner B. E., Spence A. C., Cameron A. J., Hnatiuk J. A., Ukoumunne O. C., Gold L., Abbott G., Hesketh K. D. (2013). A parent-focused intervention to reduce infant obesity risk behaviors: A randomized trial. Pediatrics, 131(4), 652–660. 10.1542/peds.2012-2576 23460688

[jsae063-B8] Caprio S. , Santoro N., Weiss R. (2020). Childhood obesity and the associated rise in cardiometabolic complications. Nature Metabolism, 2(3), 223–232. 10.1038/s42255-020-0183-z PMC9425367 32694781

[jsae063-B9] Daniels L. A. , Mallan K. M., Battistutta D., Nicholson J. M., Perry R., Magarey A. (2012). Evaluation of an intervention to promote protective infant feeding practices to prevent childhood obesity: Outcomes of the NOURISH RCT at 14 months of age and 6 months post the first of two intervention modules. International Journal of Obesity (2005), 36(10), 1292–1298. 10.1038/ijo.2012.96 22710926

[jsae063-B10] Davies W. H. , Ackerman L. K., Davies C. M., Vannatta K., Noll R. B. (2007). About your child’s eating: Factor structure and psychometric properties of a feeding relationship measure. Eating Behaviors, 8(4), 457–463. 10.1016/j.eatbeh.2007.01.001 17950934

[jsae063-B11] Dempster A. P. , Laird N. M., Rubin D. B. (1977). Maximum likelihood from incomplete data via the EM algorithm. Journal of the Royal Statistical Society, Series B (Methodological), 39(1), 1–22. 10.2307/2984875

[jsae063-B12] Dickens E. , Ogden J. (2014). The role of parental control and modelling in predicting a child’s diet and relationship with food after they leave home. A prospective study. Appetite, 76(00), 23–29. 10.1016/j.appet.2014.01.013 24480669

[jsae063-B13] DiSantis K. I. , Hodges E. A., Johnson S. L., Fisher J. O. (2011). The role of responsive feeding in overweight during infancy and toddlerhood: A systematic review. International Journal of Obesity (2005), 35(4), 480–492. 10.1038/ijo.2011.3 21427696 PMC6598438

[jsae063-B14] Draxten M. , Fulkerson J. A., Friend S., Flattum C. F., Schow R. (2014). Parental role modeling of fruits and vegetables at meals and snacks is associated with children’s adequate consumption. Appetite, 78(00), 1–7. 10.1016/j.appet.2014.02.017 24630934 PMC4034448

[jsae063-B15] Fisher J. , Sinton M. M., Birch L. L. (2009). Early parental influence and risk for the emergence of disordered eating. In Thompson L. S. J. K. (Ed.), Body image, eating disorders, and obesity in youth: Assessment, prevention, and treatment (2nd ed., pp. 17–33). American Psychological Association.

[jsae063-B16] Gelmini A. , Morawska A. (2013). Anxiety and Confidence Scale. Parenting and Family Support Centre, Australia.

[jsae063-B17] Gelmini A. , Morawska A. (2014a). *Family Lifestyle Scale*. Parenting and Family Support Centre, Australia.

[jsae063-B18] Gelmini A. , Morawska A. (2014b). Mealtime scenarios. Parenting and Family Support Centre, Australia.

[jsae063-B19] Gibbs B. G. , Forste R. (2014). Socioeconomic status, infant feeding practices, and early childhood obesity. Pediatric Obesity, 9(2), 135–146. 10.1111/j.2047-6310.2013.00155.x 23554385

[jsae063-B20] Gottschall A. C. , West S. G., Enders C. K. (2012). A comparison of item-level and scale-level multiple imputation for questionnaire batteries. Multivariate Behavioral Research, 47(1), 1–25.

[jsae063-B21] Haynos A. F. , O’Donohue W. T. (2012). Universal childhood and adolescent obesity prevention programs: Review and critical analysis. Clinical Psychology Review, 32(5), 383–399. 10.1016/j.cpr.2011.09.006 22681912

[jsae063-B22] Karlsson J. , Persson L. O., Sjöström L., Sullivan M. (2000). Psychometric properties and factor structure of the Three-Factor Eating Questionnaire (TFEQ) in obese men and women. Results from the Swedish Obese Subjects (SOS) study. International Journal of Obesity and Related Metabolic Disorders: Journal of the International Association for the Study of Obesity, 24(12), 1715–1725. 11126230 10.1038/sj.ijo.0801442

[jsae063-B23] Koh G. A. , Scott J. A., Woodman R. J., Kim S. W., Daniels L. A., Magarey A. M. (2014). Maternal feeding self-efficacy and fruit and vegetable intakes in infants. Results from the SAIDI study. Appetite, 81(00), 44–51. 10.1016/j.appet.2014.06.008 24911620

[jsae063-B24] Koplin J. J. , Kerr J. A., Lodge C., Garner C., Dharmage S. C., Wake M., Allen K. J. (2019). Infant and young child feeding interventions targeting overweight and obesity: A narrative review. Obesity Reviews: An Official Journal of the International Association for the Study of Obesity, 20(Suppl 1), 31–44. 10.1111/obr.12798 31419047

[jsae063-B25] Laws R. , Campbell K. J., van der Pligt P., Russell G., Ball K., Lynch J., Crawford D., Taylor R., Askew D., Denney-Wilson E. (2014). The impact of interventions to prevent obesity or improve obesity related behaviours in children (0–5 years) from socioeconomically disadvantaged and/or indigenous families: A systematic review. BMC Public Health, 14(1), 779. 25084804 10.1186/1471-2458-14-779 PMC4137086

[jsae063-B26] Lobstein T. , Jackson-Leach R., Moodie M. L., Hall K. D., Gortmaker S. L., Swinburn B. A., James W. P. T., Wang Y., McPherson K. (2015). Child and adolescent obesity: Part of a bigger picture. Lancet (London, England), 385(9986), 2510–2520. 10.1016/S0140-6736(14)61746-3 25703114 PMC4594797

[jsae063-B27] Matvienko-Sikar K. , Toomey E., Delaney L., Flannery C., McHugh S., McSharry J., Byrne M., Queally M., Heary C., Kearney P. M. (2019). Behaviour change techniques and theory use in healthcare professional-delivered infant feeding interventions to prevent childhood obesity: A systematic review. Health Psychology Review, 13(3), 277–294. 10.1080/17437199.2019.1605838 30991891

[jsae063-B29] Morandi A. , Meyre D., Lobbens S., Kleinman K., Kaakinen M., Rifas-Shiman S. L., Vatin V., Gaget S., Pouta A., Hartikainen A.-L., Laitinen J., Ruokonen A., Das S., Khan A. A., Elliott P., Maffeis C., Gillman M. W., Järvelin M.-R., Froguel P. (2012). Estimation of newborn risk for child or adolescent obesity: Lessons from longitudinal birth cohorts. PloS One, 7(11), e49919. 10.1371/journal.pone.0049919 23209618 PMC3509134

[jsae063-B31] Morawska A. , Sanders M. R. (2011). Parental use of time out revisited: A useful or harmful parenting strategy? Journal of Child and Family Studies, 20(1), 1–8.

[jsae063-B32] Morawska A. , Sanders M. R. (2014). Baby healthy living: Parent discussion group workbook. Parenting and Family Support Centre, The University of Queensland.

[jsae063-B33] Morris S. B. (2007). Estimating effect sizes from the pretest-posttest-control group designs. Organizational Research Methods, 11(2), 364–386.

[jsae063-B34] Neumark-Sztainer D. , Wall M., Story M., Fulkerson J. A. (2004). Are family meal patterns associated with disordered eating behaviors among adolescents? The Journal of Adolescent Health: Official Publication of the Society for Adolescent Medicine, 35(5), 350–359. 10.1016/j.jadohealth.2004.01.004 15488428

[jsae063-B35] Ogden C. L. , Fryar C. D., Martin C. B., Freedman D. S., Carroll M. D., Gu Q., Hales C. M. (2020). Trends in obesity prevalence by race and Hispanic origin—1999–2000 to 2017–2018. JAMA: The Journal of the American Medical Association, 324(12), 1208–1210. 10.1001/jama.2020.14590 32857101 PMC7455882

[jsae063-B36] Ong K. K. , Loos R. J. (2006). Rapid infancy weight gain and subsequent obesity: Systematic reviews and hopeful suggestions. Acta Paediatrica (Oslo, Norway: 1992), 95(8), 904–908. 10.1080/08035250600719754 16882560

[jsae063-B37] Paul I. M. , Savage J. S., Anzman-Frasca S., Marini M. E., Mindell J. A., Birch L. L. (2016). INSIGHT responsive parenting intervention and infant sleep. Pediatrics, 138(1), e20160762. 10.1542/peds.2016-0762 27354460 PMC4925087

[jsae063-B38] Pesch M. H. , Lumeng J. C. (2021). Early childhood obesity: A developmental perspective. Annual Review of Developmental Psychology, 3(1), 207–228. https://doi.org/10.1146.050620-124758

[jsae063-B39] Redsell S. A. , Edmonds B., Swift J. A., Siriwardena A. N., Weng S., Nathan D., Glazebrook C. (2015). Systematic review of randomised controlled trials of interventions that aim to reduce the risk, either directly or indirectly, of overweight and obesity in infancy and early childhood. Maternal & Child Nutrition, 12(1), 24–38. 10.1111/mcn.12184 25894857 PMC5029770

[jsae063-B40] Redsell S. A. , Slater V., Rose J., Olander E. K., Matvienko-Sikar K. (2021). Barriers and enablers to caregivers’ responsive feeding behaviour: A systematic review to inform childhood obesity prevention. Obesity Reviews: An Official Journal of the International Association for the Study of Obesity, 22(7), e13228. 10.1111/obr.13228 33779040

[jsae063-B41] Reilly J. J. , Armstrong J., Dorosty A. R., Emmett P. M., Ness A., Rogers I., Steer C., Sherriff A., Avon Longitudinal Study of Parents and Children Study Team. (2005). Early life risk factors for obesity in childhood: Cohort study. BMJ (Clinical Research ed.), 330(7504), 1357–1359. 10.1136/bmj.38470.670903.E0 PMC558282 15908441

[jsae063-B42] Reilly J. J. , Kelly J. (2011). Long-term impact of overweight and obesity in childhood and adolescence on morbidity and premature mortality in adulthood: Systematic review. International Journal of Obesity (2005), 35(7), 891–898. 20975725 10.1038/ijo.2010.222

[jsae063-B43] Rossiter C. , Cheng H., Appleton J., Campbell K. J., Denney-Wilson E. (2021). Addressing obesity in the first 1000 days in high risk infants: Systematic review. Maternal & Child Nutrition, 17(3), e13178. 10.1111/mcn.13178 33780128 PMC8189222

[jsae063-B44] Sanders M. R. , Kirby J. N., Tellegen C. L., Day J. J. (2014). The Triple P-Positive Parenting Program: A systematic review and meta-analysis of a multi-level system of parenting support. Clinical Psychology Review, 34(4), 337–357. https://doi.org/ 10.1016/j.cpr.2014.04.003 24842549 10.1016/j.cpr.2014.04.003

[jsae063-B45] Sanders M. R. , Markie-Dadds C., Turner K. M. T. (2001). Practitioner’s manual for Standard Triple P. Australian Academic Press.

[jsae063-B46] Sanders M. R. , Mazzucchelli T. G. (2022). Mechanisms of change in population-based parenting interventions for children and adolescents. Journal of Clinical Child and Adolescent Psychology: The Official Journal for the Society of Clinical Child and Adolescent Psychology, American Psychological Association, Division 53, 51(3), 277–294. 10.1080/15374416.2022.2025598 35133932

[jsae063-B47] Sanders M. R. , Morawska A. (2010). *Family background questionnaire*. Parenting and Family Support Centre, Brisbane.

[jsae063-B48] Schrempft S. , Van Jaarsveld C. H. M., Fisher A., Wardle J. (2015). The obesogenic quality of the home environment: Associations with diet, physical activity, TV viewing, and BMI in preschool children. PloS One, 10(8), e0134490. 10.1371/journal.pone.0134490 26248313 PMC4527827

[jsae063-B49] Smith J. A. , Saltzman J. A., Dev D. A. (2022). Mealtime emotional climate and child health: A systematic review. Eating Behaviors, 44, 101582. 34952335 10.1016/j.eatbeh.2021.101582

[jsae063-B50] Teti D. M. , Gelfand D. M. (1991). Behavioral competence among mothers of infants in the first year: The mediational role of maternal self-efficacy. Child Development, 62(5), 918–929. 10.2307/1131143 1756667

[jsae063-B51] Thompson A. L. , Adair L. S., Bentley M. E. (2013). Pressuring and restrictive feeding styles influence infant feeding and size among a low-income African-American sample. Obesity (Silver Spring, Md.), 21(3), 562–571. 10.1002/oby.20091 23592664 PMC3630475

[jsae063-B52] Thompson A. L. , Mendez M. A., Borja J. B., Adair L. S., Zimmer C. R., Bentley M. E. (2009). Development and validation of the Infant Feeding Style Questionnaire. Appetite, 53(2), 210–221. 10.1016/j.appet.2009.06.010 19576254 PMC3130353

[jsae063-B53] Walsh A. D. , Lioret S., Cameron A. J., Hesketh K. D., McNaughton S. A., Crawford D., Campbell K. J. (2014). The effect of an early childhood obesity intervention on father’s obesity risk behaviors: The Melbourne InFANT Program. The International Journal of Behavioral Nutrition and Physical Activity, 11(1), 18. 10.1186/1479-5868-11-18 24524293 PMC3928912

[jsae063-B54] Wansink B. , Kleef E. (2014). Dinner rituals that correlate with child and adult BMI. Obesity (Silver Spring, Md.), 22(5), E91–E95. 10.1002/oby.20629 24123987

[jsae063-B55] Wood C. T. , Perreira K. M., Perrin E. M., Yin H. S., Rothman R. L., Sanders L. M., Delamater A. M., Bentley M. E., Bronaugh A. B., Thompson A. L. (2016). Confirmatory factor analysis of the Infant Feeding Styles Questionnaire in Latino families. Appetite, 100, 118–125. 26876910 10.1016/j.appet.2016.02.018 PMC4799737

[jsae063-B56] World Health Organization. (2006). WHO child growth standards. Length/height-for-age, weight-for-age, weight-for-length, weight-for-height and body mass index-for-age. Methods and development. World Health Organization.

[jsae063-B57] World Health Organization. (2000). Obesity: Preventing and managing the global epidemic: Report of a WHO Consultation. World Health Organization. 11234459

